# Dental Health and Survival Following Surgery for Esophageal Cancer

**DOI:** 10.1245/s10434-026-19101-6

**Published:** 2026-01-28

**Authors:** Kenzo Buchli, Eivind Gottlieb-Vedi, Fredrik Mattsson, Ellinor Wiström, Björn Klinge, Pernilla Lagergren, Jesper Lagergren

**Affiliations:** 1 Upper Gastrointestinal Surgery, Department of Molecular Medicine and Surgery, Karolinska Institutet, and Karolinska University Hospital, Stockholm, Sweden; 2 Faculty of Odontology, Malmö University, Malmö, Sweden; 3 Department of Dental Medicine, Karolinska Institutet, Solna, Sweden; 4 Surgical Care Science, Department of Molecular Medicine and Surgery, Karolinska Institutet, Karolinska University Hospital, Stockholm, Sweden; 5 Department of Surgery and Cancer, Imperial College London, London, UK; 6 School of Cancer and Pharmaceutical Sciences, King’s College London, and Guy’s and St Thomas’ NHS Foundation Trust, London, UK

**Keywords:** Esophageal neoplasm, Teeth, Socio-economy, Prognosis

## Abstract

**Background:**

Most patients who undergo esophagectomy for esophageal cancer develop tumor recurrence and die within 5 years of surgery. An impact of preoperative dental status on survival in this patient group has been suggested but remains uncertain.

**Methods:**

This national Swedish cohort study included 871 patients who underwent esophagectomy for esophageal cancer (adenocarcinoma or squamous cell carcinoma) between 2011 and 2020 and were followed up until 2024. The exposure was the number of remaining teeth, based on dentist visits within 5 years before the esophagectomy, categorized into five (quintiles) and 10 (deciles) groups. The main outcome was all-cause 5-year mortality. Data were retrieved from medical records and national complete registries. Multivariable Cox regression provided hazard ratios (HRs) with 95% confidence intervals (CIs), adjusted for age, sex, comorbidity, tumor histology, neoadjuvant therapy, education level, and pathological tumor stage.

**Results:**

There were no statistically significant associations between the number of remaining teeth and the risk of all-cause 5-year mortality, independent of the categorization of the number of teeth. The HR was 1.13 (95% CI 0.85–1.51) comparing the lowest quintile of remaining teeth (*n* = 0–19) with the highest quintile (*n* = 29–32), and the HR was 0.98 (95% CI 0.64–1.52) comparing the lowest decile of remaining teeth (*n* = 0–10) with the highest decile (*n* = 31–32). There were no statistically significant associations in any of the subgroup analyses of age, comorbidity, or education.

**Conclusion:**

This study did not identify any association between the number of remaining teeth and the risk of 5 year mortality in patients who underwent esophagectomy for esophageal cancer.

Esophageal cancer is the seventh most common cancer globally, causing approximately 450,000 deaths annually.^1^ The mainstay of curative treatment is surgical resection (esophagectomy), which is often combined with chemo(radio)therapy. Still, the tumor recurrence rate is high, and the 5 year survival after such extensive treatment is below 50%.^2–5^ The strongest prognostic factor is tumor stage, but other factors may also influence survival, including age, sex, education, comorbidity, tumor histology, and chemo(radio)therapy.^6–10^ Another potential prognostic factor may be dental health, which is often measured objectively by the number of remaining teeth. Tooth loss, for which periodontal disease is a leading cause,^11^ has been linked with decreased life expectancy in general, which may reflect an increased prevalence of underlying comorbidities.^12^ Each increment of 10 missing teeth has been associated with a 15% increased risk of all-cause mortality, suggesting that dental status is a marker of overall health.^13^ It has also been proposed that the bacterium Porphyromonas gingivalis, the main etiological factor in the development of periodontal disease,^14^ is associated with a higher risk of esophageal cancer as well as a poor response to chemo(radio)therapy, which in turn reduces the survival.^15^ Besides promoting cancer development, this bacterium has been linked to tumor cell proliferation and stimulates tumor spread in various cancer subtypes.^16^ As such, patients with poor dental health and esophageal cancer might be at higher risk of recurrence after surgery, and as such have poorer postoperative survival. However, the literature examining the association between dental health and survival in esophageal cancer consists of only two small single-center studies. Both these studies found an association between poor dental health and worse postoperative survival in esophageal cancer,^17,18^ but the existing evidence is insufficient for making any robust conclusions.

The aim of this study was to test the hypothesis that poorer preoperative dental status, measured as a lower number of remaining teeth and investigated in a national Swedish cohort study, is associated with worse survival after esophagectomy for esophageal cancer.

## Methods

### Study design

This was a population-based cohort study of patients who underwent esophagectomy for esophageal adenocarcinoma or squamous cell carcinoma between 2011 and 2020 in Sweden, with follow-up until May 15, 2024. The association between the number of remaining teeth and the risk of mortality was studied using information collected from medical records and national health data registries. The Swedish personal identity numbers, assigned to each resident at birth or immigration, were used to link individuals’ data from each registry and to retrieve the medical records. The study was approved by the Regional Ethical Review Board in Stockholm (2024-02102-02).

### Cohort

The study was based on the Swedish Esophageal Cancer Surgery Study (SESS), a national cohort of almost all patients (98%) having undergone esophagectomy for esophageal cancer in Sweden from 1987 onwards. Earlier versions of this cohort have been presented in detail elsewhere.^7,19^ In brief, patients with esophageal adenocarcinoma (including cardia Siewert type 1 and 2) or esophageal squamous cell carcinoma were identified in the Swedish Cancer Registry, which is at least 98% complete in this regard.^20^ The Swedish Patient Registry was used to identify patients who had undergone esophagectomy, for which the registry has a 99.6% positive predictive value.^21^ The Patient Registry also provided information on age, sex, and comorbidity. The Swedish Dental Registry was used to collect information on the number of remaining teeth and dates of dentist inspections.^22^ The positive predictive value of having 1–31 intact teeth is 91.5% in this registry but lower for the extreme values of 0 remaining teeth and 32 remaining teeth.^23^ The Longitudinal integration database for health insurance and labor market studies provided data on education, used as the measurement of socioeconomic status, which is 98% complete and 85% accurate.^24^ The Swedish Cause of Death Registry provided data on mortality with 100% completeness for date of death and 98% completeness for cause of death.^25^ The medical records of all study patients were reviewed to validate the information retrieved from the registries and to add clinical information regarding chemo(radio)therapy, tumor histology, and pathological tumor stage.

### Exposures

The exposure was the number of remaining teeth preoperatively, determined from the most recent dentist visit within 5 years before the esophagectomy. The number of remaining teeth was categorized in three ways: (1) five groups as equally sized as possible, i.e., quintiles, which was the main exposure; (2) 10 groups, i.e., deciles; and (3) two groups, i.e., quintiles 1–3 and quintiles 4–5. The group with the highest number of remaining teeth was used as the reference group in all categorizations.

### Outcomes

Four mortality outcomes were analyzed: (1) all-cause 5-year mortality (the main outcome), (2) all-cause 1-year mortality, (3) all-cause 3-year mortality, and (4) disease-specific 5-year mortality. All-cause mortality was defined as any death, independent of the reason. Disease-specific mortality was defined as death with esophageal cancer as the main or contributing cause of death.

### Statistical analysis

Descriptive data of baseline variables were presented as absolute numbers with frequencies for categorical variables and as median values with interquartile ranges for the continuous variable age. Multivariable Cox regression was used to calculate hazard ratios (HRs) with 95% confidence intervals (CIs). Person–time at risk was counted from the date of esophagectomy until date of death or the end of the study (May 15, 2024), whichever occurred first. The proportional hazards assumption was examined by calculating the correlations between Schoenfeld residuals for the covariates and the ranking of individual failure times. The correlations were low and the p-values greater than the significance level of alpha 0.05, indicating that the proportional hazards assumption was met for all analyses. Four models were analyzed, as follows.

1. In a crude model, no adjustment was made.
2. In a partially adjusted model, adjustments were made for the following five confounders (with categorization in brackets): age (continuous), sex (female or male), comorbidity (Charlson Comorbidity Index score 0, 1, or ≥ 2, excluding the esophageal cancer), tumor histology (adenocarcinoma or squamous cell carcinoma), and neoadjuvant chemo(radio)therapy (yes or no).
3. In the main adjusted model, we adjusted for the five variables above and added adjustment for education ( ≤ 9 years, 10–12 years, or ≥ 13 years of formal education) to assess the relative importance specifically of education on the risk estimates.
4. In an exploratory model, pathological tumor stage (8^th^ edition American Joint Committee on Cancer/Union for International Cancer Control) was instead added (stage 0, I, II, III, or IV) to the main model. The main model did not incorporate pathological tumor stage because fewer remaining teeth may be linked to higher tumor stage, via the bacterium Porphyromonas gingivalis [26, 27]. As such, tumor stage might act as a mediating variable in the exposure–outcome pathway.

To avoid multiple testing and thus the risk of chance errors, the secondary exposures were assessed only in the main model and for the main outcome. In a sensitivity analysis, patients with 0 or 32 remaining teeth (*n* = 67) were excluded to assess potential bias due to inferior data quality of the exposure. We evaluated the possible influence of three potential effect modifiers: Age ( < 69 and ≥ 69 years), comorbidity (Charlson Comorbidity Index score 0–1 and ≥ 2), and education ( < 13 and ≥ 13 years). This was done by introducing an interaction term in the main multivariable model one by one, where HRs were derived within each stratum. Three likelihood-ratio tests were performed, and all were statistically non-significant at the alpha level of 0.05. The sensitivity analyses and effect modification analyses were performed using the main adjusted model and only for the main outcome to reduce the number of tests. Complete case analysis was conducted because the frequencies of missing data for covariates were low. The statistical analyses were conducted using the software Stata (version 16.1).

## Results

### Patients

Of 1453 eligible patients during the study period, 871 (60%) had data on the number of remaining teeth within the last 5 years before esophagectomy and were thus included in this study. Patient characteristics by groups (quintile categories) of remaining teeth are presented in Table 1. The distribution of sex and age did not differ much between the five exposure groups. The groups with fewer remaining teeth had shorter education, more comorbidities, less frequently received neoadjuvant chemo(radio)therapy, and higher rates of adenocarcinoma histology than did groups with more remaining teeth. In contrast, no consistent differences were found for pathological tumor stage.

**Table 1:** Characteristics of study patients who underwent surgery for esophageal cancer across five levels (quintiles) of remaining teeth

Remaining teeth, quintiles (range)	1 (29–32)	2 (28)	3 (25–27)	4 (20–24)	5 (0–19)
Total	153	100	223	203	192
Sex					
Male	126 (82)	83 (83)	181 (81)	154 (76)	163 (85)
Female	27 (18)	17 (17)	42 (19)	49 (24)	29 (15)
Age, years	64 (57–70)	67 (62–74)	68 (63–73)	69 (64–74)	69 (65–75)
Charlson comorbidity index score					
0	93 (61)	58 (58)	115 (52)	108 (53)	83 (43)
1	46 (30)	24 (24)	62 (28)	53 (26)	70 (36)
2	14 (9)	18 (18)	46 (21)	42 (21)	39 (20)
Neoadjuvant chemo(radio)therapy					
No	23 (15)	18 (18)	48 (22)	47 (23)	59 (31)
Yes	130 (85)	82 (82)	175 (78)	156 (77)	133 (69)
Histology					
Adenocarcinoma	123 (80)	83 (83)	183 (82)	163 (80)	167 (87)
Squamous cell carcinoma	30 (20)	17 (17)	40 (18)	40 (20)	25 (13)
Years of education					
≤ 9	32 (21)	19 (19)	64 (29)	67 (33)	73 (38)
10–12	68 (44)	45 (45)	92 (41)	96 (47)	81 (42)
≥ 13	53 (35)	36 (36)	67 (30)	40 (20)	38 (20)
Pathological tumor stage					
0	0 (0)	0 (0)	1 (0.4)	2 (1)	4 (2)
1	53 (35)	35 (35)	82 (37)	62 (31)	61 (32)
2	20 (13)	18 (18)	35 (16)	39 (19)	33 (17)
3	46 (30)	31 (31)	65 (29)	62 (31)	69 (36)
4	34 (22)	16 (16)	40 (18)	38 (19)	25 (13)

Data are presented as n (percentage) or median (interquartile range)

### Risk of mortality

There were no statistically significant associations between the number of remaining teeth and the risk of any of the mortality outcomes, independent of whether the patients were grouped into five exposure groups (quintiles), 10 groups (deciles), or two groups (quintiles 4–5 and 1–3) (Tables 2, 3, 4 ). The results did not differ much between the four statistical models. When comparing the quintile with the fewest teeth with the quintile with the most teeth, the HR of all-cause 5 year mortality was 1.19 (95% CI 0.90–1.58) in the crude model and 1.13 (95% CI 0.85–1.51) in the main model (Table 2). The corresponding HR for all-cause 5-year mortality was 1.09 (95% CI 0.72–1.67) and 0.98 (95% CI 0.64–1.52) comparing the lowest decile of remaining teeth with the highest decile in the crude and main model, respectively (Table 3). The crude and adjusted HR of all-cause 5 year mortality was 1.14 (95% CI 0.96–1.37) and 1.13 (95% CI 0.94–1.35) in the dichotomized exposure when comparing the categories with the fewest and highest number of remaining teeth (Table 4). The sensitivity analysis excluding patients with 0 and 32 teeth did not change the results (Table 2). The stratified analyses by subgroups of age, comorbidity, and education did not show any statistically significant associations regarding quintiles of the number of remaining teeth and the risk of all-cause 5-year mortality (Table 5).

**Table 2:** Risk of mortality outcomes in study patients who have undergone surgery for esophageal cancer across five levels (quintiles) of remaining teeth before surgery

Remaining teeth, quintiles (range)	1 (29–32)	2 (28)	3 (25–27)	4 (20–24)	5 (0–19)
All-cause 1 year mortality					
Crude model	1.00 (reference)	1.04 (0.61–1.79) *p* = 0.88	1.17 (0.76–1.80) *p* = 0.47	1.15 (0.74–1.79) *p* = 0.54	1.19 (0.77–1.86) *p* = 0.44
Partially adjusted model^a^	1.00 (reference)	1.01 (0.59–1.74) *p* = 0.97	1.11 (0.72–1.72) *p* = 0.64	1.11 (0.71–1.74) *p* = 0.65	1.05 (0.67–1.65) *p* = 0.83
Fully adjusted model^b^	1.00 (reference)	1.00 (0.58–1.72) *p* = 0.99	1.12 (0.72–1.73) *p* = 0.62	1.13 (0.72–1.77) *p* = 0.60	1.09 (0.69–1.72) *p* = 0.71
Exploratory model^c^	1.00 (reference)	1.05 (0.61–1.81) *p* = 0.87	1.14 (0.73–1.77) *p* = 0.56	1.11 (0.70–1.74) *p* = 0.66	1.18 (0.74–1.87) *p* = 0.48
All-cause 3 year mortality					
Crude model	1.00 (reference)	0.96 (0.66–1.39) *p* = 0.82	1.11 (0.82–1.48) *p* = 0.51	1.06 (0.78–1.43) *p* = 0.72	1.15 (0.85–1.56) *p* = 0.37
Partially adjusted model^a^	1.00 (reference)	0.95 (0.65–1.38) *p* = 0.77	1.08 (0.80–1.46) *p* = 0.60	1.06 (0.78–1.45) *p* = 0.71	1.08 (0.79–1.48) *p* = 0.62
Fully adjusted model^b^	1.00 (reference)	0.94 (0.65–1.37) *p* = 0.76	1.09 (0.80–1.47) *p* = 0.59	1.07 (0.78–1.46) *p* = 0.68	1.09 (0.80–1.49) *p* = 0.60
Exploratory model^c^	1.00 (reference)	0.99 (0.68–1.45) *p* = 0.97	1.11 (0.83–1.51) *p* = 0.48	1.07 (0.78–1.46) *p* = 0.68	1.14 (0.83–1.57) *p* = 0.41
All-cause 5 year mortality					
Crude model	1.00 (reference)	0.95 (0.67–1.34) *p* = 0.77	1.02 (0.77–1.34) *p* = 0.91	1.09 (0.83–1.44) *p* = 0.54	1.19 (0.90–1.58) *p* = 0.21
Partially adjusted model^a^	1.00 (reference)	0.94 (0.66–1.32) *p* = 0.71	1.00 (0.75–1.32) *p* = 0.97	1.09 (0.82–1.45) *p* = 0.56	1.12 (0.84–1.50) *p* = 0.42
Fully adjusted model^b^	1.00 (reference)	0.93 (0.66–1.32) *p* = 0.70	1.00 (0.75–1.32) *p* = 0.97	1.09 (0.82–1.45) *p* = 0.56	1.13 (0.85–1.51) *p* = 0.41
Exploratory model^c^	1.00 (reference)	0.97 (0.68–1.37) *p* = 0.85	1.03 (0.78–1.37) *p* = 0.83	1.08 (0.81–1.44) *p* = 0.60	1.15 (0.86–1.54) *p* = 0.34
Sensitivity analysis^d^	1.00 (reference)	0.92 (0.67–1.26) *p* = 0.60	0.97 (0.69–1.37) *p* = 0.86	1.05 (0.77–1.44) *p* = 0.75	1.13 (0.82–1.55) *p* = 0.46
Disease-specific 5-year mortality					
Crude model	1.00 (reference)	1.00 (0.70–1.42) *p* = 0.99	1.01 (0.75–1.35) *p* = 0.96	1.11 (0.83–1.48) *p* = 0.48	1.14 (0.85–1.52) *p* = 0.39
Partially adjusted model^a^	1.00 (reference)	0.99 (0.69–1.42) *p* = 0.96	1.00 (0.74–1.34) *p* = 0.99	1.12 (0.83–1.51) *p* = 0.45	1.08 (0.80–1.46) *p* = 0.60
Fully adjusted model^b^	1.00 (reference)	0.99 (0.69–1.41) *p* = 0.95	1.00 (0.75–1.34) *p* = 1.00	1.13 (0.84–1.52) *p* = 0.42	1.09 (0.81–1.48) *p* = 0.57
Exploratory model^c^	1.00 (reference)	1.03 (0.72–1.48) *p* = 0.87	1.04 (0.77–1.39) *p* = 0.80	1.12 (0.83–1.51) *p* = 0.45	1.12 (0.83–1.52) *p* = 0.45

Data are presented as hazard ratio (95% confidence interval) p-value

^a^Adjusted for sex, age, comorbidity, neoadjuvant chemo(radio)therapy, and tumor histology

^b^Adjusted for all variables in step 1 + education

^c^Adjusted for all variables in step 2 + pathological tumor stage

^d^Adjusted for all variables in step 2, excluding patients with 0 and 32 teeth

**Table 3:** Risk of all-cause 5 year mortality in study patients who underwent surgery for esophageal cancer across 10 levels (deciles) of remaining teeth before surgery

Remaining teeth, deciles (range)	Crude model	Partially adjusted model^a^	Fully adjusted model^b^	Exploratory model^c^
1 (31–32)	1.00 (reference)	1.00 (reference)	1.00 (reference)	1.00 (reference)
2 (29–30)	0.93 (0.61–1.43) *p* = 0.74	0.88 (0.57–1.35) *p* = 0.56	0.88 (0.57–1.35) *p* = 0.56	0.94 (0.61–1.45) *p* = 0.78
3 (28)	0.91 (0.60–1.39) *p* = 0.67	0.87 (0.57–1.33) *p* = 0.52	0.87 (0.57–1.32) *p* = 0.51	0.94 (0.61–1.43) *p* = 0.76
4 (27)	0.95 (0.63–1.44) *p* = 0.81	0.91 (0.60–1.39) *p* = 0.67	0.91 (0.60–1.39) *p* = 0.67	1.10 (0.72–1.68) *p* = 0.65
5 (26)	0.95 (0.60–1.51) *p* = 0.83	0.87 (0.55–1.40) *p* = 0.58	0.88 (0.55–1.41) *p* = 0.59	0.93 (0.58–1.50) *p* = 0.77
6 (24–25)	1.17 (0.79–1.74) *p* = 0.42	1.11 (0.75–1.65) *p* = 0.61	1.11 (0.75–1.66) *p* = 0.60	1.01 (0.68–1.50) *p* = 0.96
7 (22–23)	1.03 (0.67–1.58) *p* = 0.89	1.00 (0.65–1.53) *p* = 0.98	1.00 (0.65–1.54) *p* = 1.00	1.09 (0.71–1.68) *p* = 0.70
8 (19–21)	0.96 (0.62–1.46) *p* = 0.83	0.91 (0.59–1.40) *p* = 0.67	0.91 (0.59–1.41) *p* = 0.67	0.94 (0.60–1.45) *p* = 0.77
9 (11–18)	1.18 (0.78–1.78) *p* = 0.44	1.09 (0.71–1.66) *p* = 0.70	1.09 (0.71–1.66) *p* = 0.70	1.10 (0.72–1.69) *p* = 0.65
10 (0–10)	1.09 (0.72–1.67) *p* = 0.68	0.98 (0.64–1.51) *p* = 0.93	0.98 (0.64–1.52) *p* = 0.94	1.15 (0.74–1.79) *p* = 0.54

Data are presented as hazard ratio (95% confidence interval) p-value

^a^Adjusted for sex, age, comorbidity, neoadjuvant chemo(radio)therapy, and tumor histology

^b^Adjusted for all variables in step 1 + education

^c^Adjusted for all variables in step 2 + pathological tumor stage

**Table 4:** Risk of all-cause 5-year mortality in study patients who have undergone surgery for oesophageal cancer across across 2 levels (quintile 1–3 vs 4–5) of remaining teeth before surgery

Remaining teeth, quintiles (range)	1–3 (25–32)	4–5 (0–24)
Crude model	1.00 (reference)	1.14 (0.96–1.37) *p* = 0.13
Fully adjusted model^1^	1.00 (reference)	1.13 (0.94–1.35) *p* = 0.19

Data are presented as hazard ratio (95% confidence interval) p-value

^1^Adjusted for age, sex, education, comorbidity, neoadjuvant chemo(radio)therapy, and tumor histology

**Table 5:** Risk of all-cause 5 year mortality in study patients who have undergone surgery for esophageal cancer across 5 levels (quintiles) of remaining teeth before surgery, stratified by age, comorbidity and education

Remaining teeth, quintiles (range)	1 (29–32)	2 (28)	3 (25–27)	4 (20–24)	5 (0–19)
Age, years					
< 69	1.00 (reference)	0.74 (0.46–1.20) *p* = 0.19	0.92 (0.64–1.33) *p* = 0.67	1.19 (0.82–1.73) *p* = 0.47	1.11 (0.76–1.63) *p* = 0.43
≥ 69	1.00 (reference)	1.25 (0.72–2.16) *p* = 0.34	1.13 (0.71–1.80) *p* = 0.62	1.09 (0.68–1.74) *p* = 0.77	1.21 (0.76–1.93) *p* = 0.37
Charlson comorbidity index score					
< 2	1.00 (reference)	0.92 (0.63–1.34) *p* = 0.66	0.94 (0.69–1.27) *p* = 0.68	1.04 (0.76–1.41) *p* = 0.82	1.08 (0.80–1.48) *p* = 0.61
≥ 2	1.00 (reference)	1.24 (0.46–3.35) *p* = 0.67	1.60 (0.70–3.66) *p* = 0.27	1.70 (0.73–3.93) *p* = 0.22	1.77 (0.77–4.09) *p* = 0.18
Formal education, years					
< 13	1.00 (reference)	1.10 (0.72–1.69) *p* = 0.65	1.06 (0.75–1.48) *p* = 0.76	1.16 (0.83–1.63) *p* = 0.38	1.17 (0.83–1.64) *p* = 0.36
≥ 13	1.00 (reference)	0.68 (0.37–1.24) *p* = 0.21	0.88 (0.54–1.45) *p* = 0.63	0.95 (0.54–1.65) *p* = 0.84	1.08 (0.63–1.85) *p* = 0.78

Data are presented as hazard ratio (95% confidence interval) p-value, adjusted for age, sex, comorbidity, neoadjuvant chemo(radio)therapy, tumor histology, and education

## Discussion

This study did not identify any association between the number of remaining teeth and survival after surgery for esophageal cancer. The lack of association was consistent throughout all analyses regardless of the exposure categorization, outcomes, statistical models, and subgroup analyses.

Among the strengths of this study is the population-based design, which decreases the risk of selection bias and facilitates generalizability. The accuracy of all included variables from national registries and medical records was high, resulting in a low risk of misclassification. The results were adjusted for the main prognostic factors, including key variables such as tumor stage and education, which counteract confounding. The study was substantially larger than the previous studies on the topic. Among potential limitations is that the number of remaining teeth was recorded up to 5 years before surgery, which may not accurately reflect dental health at the time of surgery, introducing the possibility of non-differential misclassification. However, any such misclassification should be limited and thus not explain the results. The number of remaining teeth may seem like a rough measurement of dental health; however, studies have found the number of remaining teeth to be a valid measure.^28^ A considerable number of patients in the source cohort lacked dental registry data within 5 years before the esophagectomy, which might have introduced some level of selection bias. However, this should not explain the findings. Despite adjustment for multiple confounders, residual confounding cannot be excluded because of the observational nature of the study. Finally, the statistical power was limited in some of the subgroup analyses.

Contrary to the results of the current study, two other studies have reported an association between poor dental or oral health and worse postoperative survival in esophageal cancer.^17,18^ A Japanese single-center cohort study of 191 patients showed that a loss of seven or more teeth was associated with a nearly 2-fold increased mortality compared with patients with a loss of fewer than seven teeth (HR 1.87; 95% CI 1.22–2.87).^17^ When a similar cut-off level was analyzed in the dichotomized analysis in the current study, a non-significant difference with an HR of 1.14 was observed. Another Japanese single-center study of 90 patients using an oral health assessment tool (OHAT), found that poorer oral health (OHAT score ≥ 3) was associated with an increased 5 year mortality compared with better scores (HR 3.38; 95% CI 1.10–10.43).^18^ Both these studies were conducted in Japan, where squamous cell carcinoma is the dominating histology of esophageal cancer, unlike in Sweden, where adenocarcinoma is the most frequent histological type of esophageal cancer. The fact that these cancer types have clearly different etiology and pathogenesis is a possible explanation for the differences in results.^29^ The previous study that assessed the number of remaining teeth did not adjust for socioeconomic status or comorbidity,^17^ and it is possible that the number of remaining teeth served as a proxy for comorbidity, an essential prognostic factor after surgery for esophageal squamous cell carcinoma.^30^ The other study assessed overall oral health rather than dental health alone, which might explain the different findings to those of the current study.^18^

In conclusion, this large, population-based study of patients who underwent esophagectomy for esophageal cancer did not identify any association between the number of remaining teeth and survival.
